# The Values of Stocks at Given Times: King Edward's Hospital Fund for London Statistics.—Fifth Notice


**Published:** 1915-02-20

**Authors:** 


### THE VALUES OF STOCKS AT GIVEN TIMES.

### King Edward's Hospital Fund for London Statistics.—Fifth Notice.*

The whole object of the Uniform System and the stimulus given for its correct working by the issue of the Statistical Report is to encourage the hospitals to prepare their figures upon as businesslike and as scientific a plan as is possible. On more than one occasion the accounts of hospitals have, in the official directions of the Central Funds, been compared with those of commercial undertakings; particularly was this so when reasons were stated why certain estimates should be made in dividing in-patient and out-patient cost.

The whole system, of course, is a progressive one, and as we gain experience of it we observe from time to time various points where, in the aim at perfection, improvements can be made. The Central Hospital Funds have shown that they are always ready to accept and consider criticism, and have more than once taken into consultation the officials upon whose shoulders it falls to work the system.

There are one or two suggestions in this connection we should like to refer to and submit to those who are best qualified to search for and advise on improvement. One of these is the question of the difference between the values of stocks of goods on January 1 and December 31 each year. If a hospital in respect of its accounts were conducted on the lines of a commercial undertaking, stocks, which represent so much money sunk, would without doubt be expressed by the accountant in his annual statements in pounds, shillings, and pence. Taking the supplies of the hospitals as a whole, the period between the times goods are received and distributed is very brief. This condition probably applies to the whole of the commissariat department, as in the aggregate the most costly of the goods handled are of a perishable nature. However, even in this department, such things as wines, spirits, household requisites, preserved foods, and other articles not required for immediate use may materially vary in the values of the stocks on the two dates named.

But if we look elsewhere we find room for a very considerable difference in values, notably in the quantity of drugs, etc., stocked from time to time in the dispensary. Especially is this the case in a year like 1914. In one of the greater hospitals it seems likely that in normal times there could very well be dispensary stocks to the value of £1,500 in hand at one time. A year ensues when prices are making rapid fluctuations; if they are on the upward move the responsible officer feels chary of buying goods which may possibly not be required for some months, as he would in prudence do if prices were apparently settled. If there is anything in this argument it appears possible that the difference in the value of these stocks in such an establishment at the beginning and the close of the year might amount to quite £500, which sum would largely represent the drugs, etc., purchased in the preceding year, on the accounts of which their cost would fall. The cost per bed and per out-patient attendance, therefore, in neither year would be a true one, and when prices had recovered their balance and stocks were again replenished the averages would again be misleading. Another department where the same arguments would apply is covered by the heading Domestic, where purchases of bedding, linen, hardware, and crockery would be kept down, under certain circumstances, to the lowest possible limits.

### Annual Cleaning.

Another point calling for close examination is in respect of the cost of annual cleaning. Of course, we do not overlook the fact that when the averages in respect of the bed and the attendance are presented establishment expenses are omitted from the calculation, but, as we have shown in previous articles, that which is represented by the cost under one department has often a bearing on another department, and, though the expenditure of that department may be removed, its influence remains. A hospital may in one year close one-third of its wards for cleaning or redecoration. (The question of whether this method of carrying out the work is desirable, although not a purely statistical consideration, we shall refer to later.) In the following year the same hospital may close the remaining two-thirds of its beds, or close no beds at all, and confine the painters' work to that part of the building used by the staff. The number of occupied beds, therefore, varies, but the whole of the standing charges of the hospital, which apply to every part of the hospital and every detail of the work, remain the same from year to year. Rent, rates, general administration cost, most domestic expenses and nursing salaries are the same whatever the number of the wards temporarily closed. It is quite conceivable that, of two hospitals worked at exactly the same expense, one will show a steady cost over a series of years, while the other will show a slightly lower cost in every year of the series except one, and a considerably higher cost in that one.

We do not think that any hospital administrator, especially if his experience has been in respect of the hospitals of the cities and larger towns, would defend any plan of cleaning which did not include the turning out of every part of a hospital annually. Should one be referring to a hospital in a position untouched by the smoke and dust of a closely populated part, this limit could be extended to a biennial cleaning. This may be called an ideal method, but it is really the only hygienic one possible, and if a hospital is painted throughout with the best materials, the annual cleaning is a simple and rapid process, and has the advantage of causing the expenditure to be divided in the only way which will render the accounts properly comparable from year to year and with the statistics of other institutions working under a similar scheme.

* Previous articles appeared on October 10 and December 12, 1914, and January 23 and February 6, 1915.

